# Costs analysis of surgical treatment of stress urinary incontinence in a brazilian public hospital, comparing Burch and synthetic sling techniques

**DOI:** 10.1590/S1677-5538.IBJU.2017.0232

**Published:** 2018

**Authors:** Leo Francisco Limberger, Fernanda Pacheco Faria, Luciana Silveira Campos, Karin Marise Jaeger Anzolch, Alexandre Fornari

**Affiliations:** 1 Serviço de Ginecologia do Hospital Nossa Senhora da Conceição, Porto Alegre, RS, Brasil; 2 Hospital Moinhos de Vento, Porto Alegre, RS, Brasil; 3 Ambulatório de Disfunções miccionais da Santa Casa de Porto Alegre, Porto Alegre, RS, Brasil

**Keywords:** Urinary Incontinence, Suburethral Slings, Costs and Cost Analysis

## Abstract

**Introduction:**

Surgical treatment of urinary incontinence progressed significantly with the introduction of synthetic slings. However, in some public Brazilian hospitals, the costs of these materials prevent their routine use.

**Objective:**

To compare the costs of ambulatory synthetic sling surgery with an historical series of patients submitted to Burch surgery in a Brazilian public hospital.

**Materials and Methods:**

Twenty nine incontinent patients were selected to synthetic sling surgery. Demographic data were prospectively collected and also the costs of the procedure, including drugs and materials, use of surgical and recovery wards, medical staff and hospitalization. These data were compared to the costs of 29 Burch surgeries performed before the introduction of synthetic slings.

**Results:**

Demographic data were similar, although median age was lower in the group submitted to Burch surgery (46.3±8.6 versus 56.2±11.3 (p<0.001)). Cost was significantly lower in patients submitted to sling in all items, except for time spent in recovery ward. Total value of 29 Burch surgeries was R$ 217.766.12, and of R$ 68.049.92 of 29 patients submitted to sling surgery (p<0.001).

**Conclusion:**

Burch surgery was more expensive than ambulatory synthetic transobturator sling surgery, even when the cost of the synthetic sling was considered.

## INTRODUCTION

Stress urinary incontinence is a quite common disease in perimenopausal women, with significant lowering of quality of life, causing social isolation, low self-stem and depression (1). Treatment is costly in the public Brazilian health system, with reduced resources to attend all population demands (2). Surgical treatment of stress urinary incontinence evolved significantly with the introduction of medium urethral slings, proposed by Petros and Ulmsted (3, 4). Today, this is the most frequent procedure for this condition in Brazil. However, in spite of higher morbidity of Burch colposuspension surgery in relation to synthetic slings (5, 6) in many public services the costs of the material prevent their routine use. The objective of the present study is to compare costs of surgical treatment of women with stress urinary incontinence comparing the use of synthetic slings and a historical series of patients submitted to Burch surgery in the same institution.

## MATERIALS AND METHODS

Twenty nine patients were selected for stress urinary incontinence surgery, consecutively, that attended the ambulatories of Gynecology and Urology of Hospital Nossa Senhora da Conceição in Porto Alegre, RS, Brazil. The inclusion criteria were: patients with 18 to 70 years old, agree to be submitted to the procedure, satisfactory clinical condition for ambulatory surgery and diagnosis of stress urinary incontinence (anamnesis, physical exam, urodynamic evaluation when indicated). The exclusion criteria were: pregnancy or recent puerperium (until six months following delivery), previous surgical treatment of urinary incontinence, active vaginal or urinary infection, previous malignant pelvic disease or radiotherapy, neurological diseases, coagulation disorders or immunological diseases. All patients signed an informed consent.

The study was approved by Ethical Committee under the number 09-214. A total of 29 patients in the sling group was considered adequate for cost-efficiency analysis, for comparison with the same number of patients submitted to Burch surgery in a historical series of our Hospital, performed right before the beginning of this protocol. Since literature presents a huge amount of articles related to the lower morbidity of synthetic slings, randomization was not performed, and it was decided to compare patients with a historical series. Patients were submitted to ambulatory conventional transobturator sling surgery under spinal anesthesia. Demographic data, time of surgical room, time of surgery, time in recovery award, work time of professionals, and drugs, materials and hospitalization costs were collected. Material values, time of surgical room, drugs and professionals in all procedures were updated by the hospital financial department to those performed in May 2014, in order to make them comparable. Costs related to material used in surgical room (surgical threads, gauzes, compresses, antisepsis, anesthetics, saline, etc.) were calculated according to materials described at room sheets available at the financial department. Anesthetic professional costs were calculated according to time in relation to medium wage of hired professionals by the institution and included all surgical time plus previous anesthetic consultant of patients submitted to Burch procedure. Surgeons costs included surgical time calculated according to medium wage of surgeons hired by the institution, plus one visit per hospitalization day of patients submitted to Burch procedure. Surgical room costs were calculated in relation to total cost of surgical ward divided by the number of rooms per hour, considering a variation for the nocturnal time (after 7 pm). In relation to drugs costs, it was considered only those used at wards and recovery room. Data were analyzed by SPSS version 16.0 software to calculate statistical significance. Normal distributed variables were analyzed by Student t test and described as medium and standard deviation, and those with not-normal distribution were analyzed by Mann-Whitney test. Statistical significance was considered when p<0.05.

## RESULTS

Demographic data are presented at Table-1. It is observed similar results of groups in relation to parity and BMI. However, age of patients was lower in Burch group (p<0.01). Time of surgical room was significantly lower in patients submitted to sling surgery (2.91±0.74 h Burch group and 0.92±0.24 h for sling group (p<0.001)) and also for surgical time (2.48±0.82h Burch group and 0.31±0.26h Sling group (p<0.001)).

**Table 1:** Demographic characteristics.

Characteristics	Burch	Sling	P*
Age	46.3±8.6	56.2±11.3	<0.001
Parity	3.27±1.7	2.68±1.07	0.158
BMI	27.8±4.2	28.5±5.4	0.590

* Student t test

Cost analysis of Burch surgery and sling procedure are presented at Table-2. In the historical series of 29 consecutive patients submitted to Burch surgery from November 2008 to March 2011, the total cost was R$ 217.766.12, and of the 29 patients submitted to sling procedure from March 2011 to May 2014 the total cost was R$ 68.049.92 (a 69% savings of R$ 149.716.20). Medium time of hospitalization of patients submitted to Burch surgery was 4.03 days (a total of 117 hospitalization days). All patients submitted to sling procedure were ambulatorial, without the need of hospitalization. General costs, and particular costs (materials, drugs, surgical time, use of surgical room, time of surgeon and anesthesiologist, hospitalization time) were converted to current prices by the institution, and showed a statistical significant benefit favoring sling procedure (p<0.01), as demonstrated at Table-2. This difference is also lower in relation to Burch group when the cost of the sling kit (around R$ 1.300.00) and time on recovery ward are considered (p<0.002).

**Table 2:** Comparative study of costs of Burch surgery x sub-urethral sling procedure.

					2014 Base Year			
Description	Total costs of 29 patients				Unitary cost per patient			
	Burch	Sling	Difference (Burch X Sling)	D%	Burch	Sling	Difference (BurchXSling)	P*
Drugs	1.353.81	69.74	1.284.07	-95%	46.68	2.40	44.28	<0.001
Surgical room material	11.631.63	5.018.22	6.613.41	-57%	401.09	173.04	228.05	<0.001
Surgical room	45.724.69	13.168.98	32.555.72	-71%	1.576.71	454.10	1.122.61	<0.001
Surgeon	10.898.46	1.030.43	9.868.03	-91%	375.81	35.53	340.28	<0.001
Anesthesiologist	15.483.74	3.522.36	11.961.38	-77%	533.92	121.46	412.46	<0.001
Recovery room	6.883.89	7.540.19	(656.30)	10%	237.38	260.01	- 22.63	0.002
Hospitalization	125.789.89	-	125.789.89	-100%	4.337.58	-	4.337.58	-
TOTAL	**217.766.12**	**30.349.92**	187.416.20	-86%	7.509.18	1.046.55	6.462.63	<0.001
SLING	-	37.700.00	(37.700.00)		-	1.300.00	- 1.300.00	-
GRAND TOTAL	**217.766.12**	**68.049.92**	**149.716.20**	-69%	7.509.18	2.346.55	5.162.63	<0.001

* Mann-Whitney test

## DISCUSSION

The results of the present study clearly demonstrate that sling procedure for stress urinary incontinence treatment is less costly than Burch surgery, even when the costs of the synthetic sling is considered. After analysis of only 29 patients it was possible to detect a saving of R$ 149.716.20. This could pay for 60 other sling surgeries. A study by Laudano et al. (7) showed similar results, and demonstrated higher cost-efficiency of TVT in relation to Burch surgery. That was a more complex study than ours, since it followed up patients for up to 10 years. However, a published meta-analysis by Rawlings and Zimmern (8) in 2016 was inconclusive to compare cost-efficiency of different surgical techniques; they affirmed that there is variation of results according to surgeons, technique and different regions. Maybe direct comparison as done in our study is the best way to answer this question. Our study did not evaluate laparoscopic Burch surgery; however, in a 2013 study by Lo et al. (9) it was observed advantage of costs of TVT in relation to laparoscopic Burch surgery.

However, direct cost is only one of the advantages demonstrated in our study. The lower surgical time of sling technique allows for better use of hospital structure for a large number of procedures. In our study, changing Burch procedure for sling technique of 29 patients allowed for more 57 available hours of surgical room, favoring treatment of this condition and eventually of others. These data are similar of Ankardal et al. (10) where the surgeon/minute costs were calculated, and it was observed a significant financial advantage of sling procedure. In that study, it was also considered that in Sweden sling procedure is performed without an auxiliary surgeon, in contrast to Burch surgery, that requires the presence of one auxiliary surgeon, increasing the costs. This is not done in our country; traditionally, a surgeon and an auxiliary professional perform sling procedure. The costs of the auxiliary surgeon were not considered in our study since it was performed in a school Hospital were residents assist surgical procedures (Burch and sling techniques).

Also, the absence of need of hospitalization allows for redistribution of hospital beds for other occasions, with higher needs, and this seems to be a global tendency (11, 12). Our study demonstrated that the treatment of 29 patients made available 117 daily hospitalizations. In our country, this is extremely important, since the number of available beds of public hospitals is insufficient to attend all demands of population. It should be pointed out that time at recovery ward was significantly longer of patients submitted to sling procedure, probably due to ambulatory characteristic of sling surgery. It should also be pointed out that patients submitted to Burch or sling surgery received spinal anesthesia, with a shorter time during sling procedure, and a counterpart higher time spent at recovery room for complete dissipation of anesthesia effects (13).

The present study did not analyzed patient's satisfaction and associated morbidity. However, many published studies clearly document lower morbidity of sling procedure in relation to Burch surgery (5, 6). When surgical results are considered, the literature is also extensive, and several guidelines demonstrate similar or better results of Sling surgery regarding long term continence and also lower level of urgency, urinary urge incontinence and de novo urinary incontinence in relation to Burch surgery (12, 14).

One of the negative aspects of the present study is the comparison with a historical series, that may introduce bias; however, the last 29 Burch surgeries were selected, right before the beginning of sling procedures. For this analysis, there was no selection, and the results were close to reality of our service: patients routinely are admitted one day prior to surgery and many times the procedure is delayed due to complications or other emergence surgeries (very common in Brazilian public health system). Therefore, hospitalization time usually is longer due to administrative reasons and not necessarily medical, and it also adds costs to the procedure (15). Burch surgery also is costly due to pre-anesthetic evaluation, performed when patient is admitted in the previous day. This cost is not observed in ambulatory procedures. Also, slings were performed in the usual time, not after 7p.m., when surgical room is costly; this was observed in some patients submitted to Burch surgery.

Another limitation of this study is that all sling procedures were performed by the same surgeon following a protocol of selection of patients suitable for the ambulatorial procedure with low risk of complications. However, we think that this protocol may be implemented in any facility that perform public health surgeries with good results. Another aspect is the low number of patients. However, even with only 29 patients in each group, it was possible to detect a significant reduction of costs; therefore, this main aspect minimizes interferences or biases.

It is clear in our study that surgical treatment of urinary incontinence by Burch technique is costly, demands more surgical time and hospitalization, and sling procedure should be preferable whenever possible. We hope that this study sensitizes public managers to allow the use of synthetic slings for routine treatment of female stress urinary incontinence, reducing costs and improving quality of service provided.
